# Hypothalamic control systems show differential gene expression during spontaneous daily torpor and fasting-induced torpor in the Djungarian hamster (*Phodopus sungorus*)

**DOI:** 10.1371/journal.pone.0186299

**Published:** 2017-10-12

**Authors:** Ceyda Cubuk, Hanna Markowsky, Annika Herwig

**Affiliations:** 1 Zoologisches Institut, Universität Hamburg, Hamburg, Germany; 2 Institut für Neurobiologie, Universität Ulm, Ulm, Germany; University of Oxford, UNITED KINGDOM

## Abstract

Djungarian hamsters are able to use spontaneous daily torpor (SDT) during the winter season as well as fasting-induced torpor (FIT) at any time of the year to cope with energetically challenging environmental conditions. Torpor is a state of severely reduced metabolism with a pronounced decrease in body temperature, which enables animals to decrease their individual energy requirements. Despite sharing common characteristics, such as reduced body mass before first torpor expression and depressed metabolism and body temperature during the torpid state, FIT and SDT differ in several physiological properties including torpor bout duration, minimal body temperature, fuel utilization and circadian organization. It remains unclear, whether SDT and FIT reflect the same phenomenon or two different physiological states. The hypothalamus has been suggested to play a key role in regulating energy balance and torpor. To uncover differences in molecular control mechanisms of torpor expression, we set out to investigate hypothalamic gene expression profiles of genes related to orexigenic (*Agrp*/*Npy*), circadian clock (*Bmal1*/*Per1*) and thyroid hormone (*Dio2*/*Mct8*) systems of animals undergoing SDT and FIT during different torpor stages. Orexigenic genes were mainly regulated during FIT and remained largely unaffected by SDT. Expression patterns of clock genes showed disturbed circadian clock rhythmicity in animals undergoing FIT, but not in animals undergoing SDT. During both, SDT and FIT, decreased *Dio2* expression was detected, indicating reduced hypothalamic T3 availability in both types of torpor. Taken together, our results provide evidence that SDT and FIT also differ in certain central control mechanisms and support the observation that animals undergoing SDT are in energetical balance, whereas animals undergoing FIT display a negative energy balance. This should be carefully taken into account when interpreting data in torpor research, especially from animal models of fasting-induced hypometabolism such as mice.

## Introduction

The use of torpor in times of energetically challenging environmental conditions is a common strategy, which has been identified in most orders of mammals. Reduction of metabolic rate and body temperature (T_b_), the two main characteristics of torpor, enable the animal to reduce energy expenditure and lower energy requirements with almost no evidence of tissue or organ damage after rewarming from the torpid state [1–7].

The Djungarian hamster (*Phodopus sungorus*, also known as Siberian hamster) uses spontaneous daily torpor (SDT) to save energy during the harsh winters of Central Asian steppes. SDT in Djungarian hamsters is the final trait of various adaptions (severe body weight loss, molt to white winter fur, gonadal regression) to the winter season [8,9]. The onset of winter acclimatization is triggered by decreasing day length during autumn, driven by modification of melatonin production in the pineal gland, and can easily be induced by adjusting the light-dark cycle in the laboratory [10–12]. SDT is controlled by the circadian clock and usually limited to the resting phase, allowing the hamsters to maintain foraging activities during the night throughout winter. The torpid state is initiated by an active metabolic depression (25% below the level of resting metabolic rate), followed by a drop in T_b_ to a minimum value of approximately 15°C, reduced ventilation, heart rate and locomotor activity with an average duration of six hours per torpor bout [3,13,14]. When SDT is used frequently, Djungarian hamsters are able to save up to 65% of total energy requirements during the winter season [15].

Food scarcity, hence energy depletion, is not restricted to the winter months, but can occur at any time of the year and is able to induce fasting-induced torpor (FIT) in many small mammals, including mice [16–20]. This form of torpor can occur not only during any season but also at any time of day. Although SDT and FIT in Djungarian hamsters both involve metabolic depression and decreased T_b_, they show some distinct physiological characteristics including differences in preparatory time period before the first torpor episode occurs, circadian control, fuel utilization as well as differences in torpor depth and duration [19,20]. The physiological variations between these two forms of torpor suggest that they might be regulated by distinct central control mechanisms.

Several hypothalamic systems have been linked to the regulation of energy balance and torpor. In the arcuate nucleus (ARC), food intake and energy expenditure are closely linked to the expression of orexigenic (*Npy*/*Agrp*) and anorexigenic (*Pomc*/*Cart*) genes. When orexigenic neuropeptides are activated, food consumption is enhanced and energy expenditure reduced [21–23]. Intracerebroventricular injections of NPY induce torpor like hypothermia in *P*. *sungorus*, likely mediated by NPYY1 receptors. This hypothermic state seems to resemble torpor patterns of FIT bouts rather than SDT bouts [24,25]. Moreover, ARC lesions by monosodium glutamate injections prevent SDT expression [26].

The suprachiasmatic nuclei (SCN) host the circadian clock, which controls the circadian organization of daily rhythms in biochemistry, physiology and behavior by autonomous transcription-translation feedback loops. These feedback loops consist of positive (BMAL1/CLOCK) and negative (PER/CRY) elements, activating and inactivating each other’s transcription in a roughly 24 hour rhythm that is synchronized to the light-dark cycle of the day [27]. SDT is clearly timed by the circadian clock and expression of clock genes remains largely rhythmic [13,28,29]. The ablation of the SCN leads to a disordered SDT onset, which is no longer restricted to the daytime, but does not prevent the expression of torpor [30]. In FIT, a circadian rhythmicity of torpor timing is absent, thus FIT can occur at any time of a day.

Thyroid hormones are known for their role in regulating energy balance by peripheral but also central mechanisms [31,32]. The hypothalamic thyroid hormone system has been shown to be a crucial driver of seasonal adaptions, including long-term shifts in energy balance [33–35]. Thyroid hormones are transported into the hypothalamus by transporters (e.g. *Mct8*) where they are activated or inactivated by the type-II-deiodinase (*Dio2*) and type-III-deiodinase (*Dio3*), respectively [36,37]. These two enzymes are co-expressed in tanycytes of the third ventricle and expression is regulated in a season dependent manner [33,35,38,39]. Moreover, T3 availability affects torpor behavior. Chemical inhibition of T3 production promotes torpor expression, whereas excess T3 in the periphery as well as locally in the hypothalamus, is able to prevent torpor in winter adapted animals [40–42].

Here we set out to investigate differences in regulatory mechanisms of SDT and FIT induced torpor. For this purpose we compared hypothalamic expression profile of *Agrp* and *Npy* (orexigenic system), *Bmal1* and *Per1* (circadian clock system) and *Dio2* and *Mct8* (thyroid hormone system) over different torpor stages of fasted and *ad libitum* fed hamsters. Alterations in the orexigenic as well as circadian system demonstrate that SDT and FIT are at least partly regulated by distinct mechanisms.

## Material and methods

### Animals

The experiments were performed in accordance with the German Animal Welfare Law and approved by the local animal welfare authorities (No. 4_16 and No. 114_14, Hamburg, Germany).

All Djungarian hamsters (*P*. *sungorus*) were descended from our own breeding colony at the Zoological Institute of the University of Hamburg, Germany. Hamsters were bred and raised under artificial long day conditions (LD) with a light-dark-cycle of 16 hours light and 8 hours dark and an ambient temperature of 21 ± 1°C. The hamsters were housed individually in plastic cages (Macrolon Type III) with free access to drinking water before and during experiments. They were fed a hamster breeding diet (Altromin 7014, Germany) *ad libitum* before experiments started.

### Experimental setup and sampling

At the age of three to five months a total number of 70 hamsters was transferred to short day conditions (SD) with a light-dark-cycle of 8 hours light and 16 hours dark and an ambient temperature of 18 ± 1°C to develop their winter phenotype (catabolic state, white winter fur, occurrence of SDT, gonadal regression). Animals not clearly showing a winter phenotype after 10 weeks of SD exposure were excluded from the experiments. A group of 20 hamsters remained under LD, representing the summer phenotype (anabolic state, brownish-grey summer fur, reproductively active). Animals were separated into three SD-groups (SDT, FIT-SD, non-torpid SD animals (NT-SD)) and one LD-group (FIT-LD). Each group consisted of 20 male and female hamsters.

After 12 weeks under SD all animals were implanted i.p. with DSI-transmitters (Model TA-F10, St. Paul, MN, USA) under isoflurane anesthesia (2.0–2.5%, Forene, Abott, Wiesbaden, Germany) and analgesia by s.c. injection of carprofen (5 mg/kg, Paracarp, IDT Biologika, Germany). The LD-group was implanted with DSI-transmitters in the same way. Surgeries were carried out as previously described [41]. Core T_b_ of each hamster was measured in three minute intervals until the end of the experiment to precisely determine the T_b_ while sampling during different torpor stages as well as to calculate torpor duration and depth.

**Fig 1:**
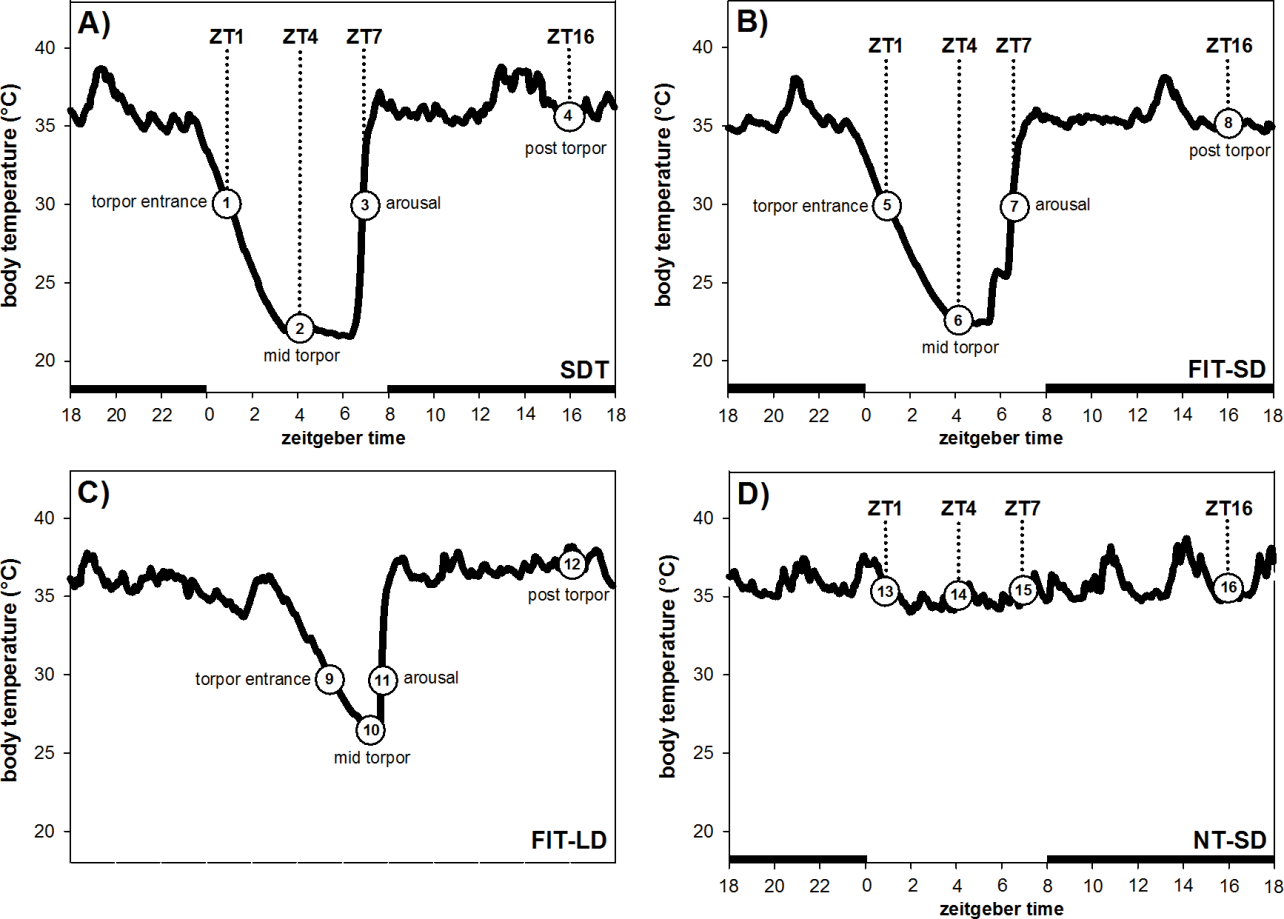
**Sampling points for hamsters undergoing SDT (A), FIT-SD (B) and FIT-LD (C) and of hamsters remaining NT-SD (D).** Black bars in the x-axis represent the dark phase of the light-dark cycle.

The three SD groups were sampled according to torpor state and Zeitgeber time (ZT, ZT0 = lights on), whereas the LD group was sampled according to torpor state only (Fig 1).

The spontaneous torpor group kept under SD (SDT) was fed *ad libitum* throughout the experiment. These animals were culled at different stages of a spontaneous daily torpor bout: during torpor entrance at ZT1 (n = 5, Tb: 30.8°C ± 0.5°C), mid torpor at ZT4 (n = 5, Tb: 22.5°C ± 1.5°C), arousal at ZT7 (n = 5, Tb: 30.4°C ± 0.4°C) and in a post torpid state at ZT16 (n = 5, Tb: 35.7°C ± 0.6°C) (Fig 1A, group 1–4; Table 1).

**Table 1:** Sampling T_b_ data of animals undergoing SDT, FIT-SD, FIT-LD and remaining NT-SD. SDT and FIT-SD were sampled according to torpor state and ZT, FIT-LD was sampled according to torpor state and NT-SD was sampled according to ZT.

	torpor entrance (ZT1)	mid torpor (ZT4)	arousal (ZT7)	post torpor (ZT16)
**SDT**	30.8°C ± 0.5°C	22.5°C ± 1.5°C	30.4°C ± 0.4°C	35.7°C ± 0.6°C
**FIT-SD**	30.1°C ± 0.4°C	21.3°C ± 0.9°C	30.3°C ± 0.5°C	35.0°C ± 2.7°C
**FIT-LD**	29.7°C ± 0.4°C	25.4°C ± 1.4°C	30.7°C ± 0.5°C	35.1°C ± 1.9°C
**NT-SD**	35.7°C ± 0.5°C	35.7°C ± 0.4°C	35.6°C ± 0.4°C	36.2°C ± 1.3°C

For the SD fasting-induced torpor group (FIT-SD), daily food consumption of each hamster was individually recorded during week 11 in SD for seven consecutive days to calculate average food intake. Throughout the experiment the hamsters were provided 60% of their daily food intake at ZT6 for five days, followed by three days of *ad libitum* feeding. The animals were weighed twice a week to monitor body weight loss. In case of a critical body weight loss over 25% of their LD body mass the hamsters were fed additional 10%. To ensure sampling during FIT bouts, we collected brain samples only after at least two days of food restriction, when the hamsters had already shown torpor bouts. Once the hamsters displayed FIT, they were culled during torpor entrance at ZT1 (n = 5, Tb: 30.1°C ± 0.4°C), mid torpor at ZT4 (n = 5, Tb: 21.3°C ± 0.9°C), arousal at ZT7 (n = 5, Tb: 30.3°C ± 0.5°C) and in a post torpid state at ZT16 (n = 5, Tb: 35.0°C ± 2.7°C) (Fig 1B, group 5–8; Table 1).

For the LD fasting-induced torpor group (FIT-LD), average daily food consumption for each hamster was determined as described for FIT-SD. The food was provided daily at ZT14. During the first experimental week hamsters were fed 30% of their daily food intake followed by four weeks with 60% feeding to induce FIT [19]. The hamsters were weighed twice a week and received additional 10% food, if they exceeded a body weight loss of over 25%. Hamsters not showing torpor within these four weeks were fed *ad libitum* again for three days, followed by four days with 30% food and one week with 60% food. Since FIT is not under circadian control, these animals were not sampled at a particular ZT, but during torpor entrance at ZT5 –ZT9 (n = 5, Tb: 29.7°C ± 0.4°C), mid torpor at ZT5 –ZT10 (n = 5, Tb: 25.4°C ± 1.4°C), arousal at ZT7 –ZT11 (n = 5, Tb: 30.7°C ± 0.5°C) and in a post torpid state at ZT20 (n = 5, Tb: 35.1°C ± 1.9°C) (Fig 1C, group 9–12; Table 1).

The SD non-torpid group (NT-SD) was fed *ad libitum* throughout the experiment. These animals were winter adapted and already spontaneously expressed torpor. They were culled on a day without torpor in a non-torpid state at ZT1 (n = 5, Tb: 35.7°C ± 0.5°C), ZT4 (n = 5, Tb: 35.7°C ± 0.4°C), ZT7 (n = 5, Tb: 35.6°C ± 0.4°C) and ZT16 (n = 5, Tb: 36.2°C ± 1.3°C) as respective control (Fig 1D, group 13–16; Table 1).

All hamsters used in this experiment were sacrificed by CO_2_ inhalation. Brains were dissected, immediately frozen on dry ice and stored at -80°C until further use.

### Isolation of total RNA and cDNA synthesis

Hypothalamic blocks were cut from frozen brain samples as described in Cubuk *et al*., 2017[43]. Hypothalamic samples were homogenized in 1 ml TriFast by using a micropestle. Total RNA was isolated using peqGOLD Trifast^TM^ (Peqlab, Erlangen, Germany) and purified by using the Crystal RNA MiniKit (Biolabproducts, Bebensee, Germany) including an on-column digestion with RNase-free DNase (Quiagen, Hilden, Germany) according to the manufacturer`s instructions. Total RNA was quantified spectrometrically, RNA purity was assessed by the 260/280 nm ratio on a NanoDrop 1000 spectrophotometer and RNA integrity was proven by formaldehyde agarose gel electrophoresis. 1 μg of total RNA of each sample was used for cDNA synthesis, carried out with the RevertAid H Minus First Strand cDNA Synthesis Kit (Thermo Scientific, Waltham, MA, USA) using oligo-(dT)-primer (0.5 μg/μl) according to manufacturer`s instructions. Total cDNA samples were stored at -20°C until usage as template for Real Time qPCR or standard plasmid generation.

### Cloning and sequencing

Standard plasmids were generated from 90–200 bp long coding sequence fragments of agouti related neuropeptide (*Agrp*), brain and muscle Arnt-like protein-1 (*Bmal1*), iodothyronine deiodinase 2 (*Dio2*), monocarboxylate transporter 8 (*Mct8*), neuropeptide Y (*Npy*), period circadian clock 1 (*Per1*), hypoxanthine phosphoribosyltransferase (*Hprt*), actin beta (*Actb*), ribosomal protein lateral stalk subunit P0 (*Rplp0*) and 18S ribosomal RNA (*Rn18s*) by gene specific primers (Table 2). All primers were designed on *P*. *sungorus* specific sequences obtained from our previous Illumina study [43]. 18–25 bp long primers were designed using the online tool OligoAnalyzer 3.1 with a melting temperature of 60°C ± 1.1°C. The amplicons were cloned into the pGEM®-T Easy Vector System (Promega, Madison, USA) according to manufacturer`s instructions and sequenced by the commercial sequencing platform GATC Biotech (Konstanz, Germany).

**Table 2:** *P*. *sungorus* specific primer sequences used for standard plasmid generation and qPCR.

gene	5´3´sequence		melting	amplicon
			temperature	length
*Agrp*	forward	GCC TTT GCC CAA CAT CCG TTG	59.8	99 bp
	reverse	GCT ACT GCC GCT TCT TCA ATG CC	60.9	
*Bmal1*	forward	GCT CAA GAG ACC CCA GGT TAT CC	59.1	145 bp
	reverse	GGC TCA TGA TGA CAG CCA TCG C	60.8	
*Dio2*	forward	TGA AGA AAC ACA GGA GCC AAG AGG A	60.0	111 bp
	reverse	CAT TAT TGT CCA TGC GGT CAG CCA	59.8	
*Mct8*	forward	GTC CTC TCA TTC CTG CTC CTG G	59.2	151 bp
	reverse	GTC CCA CCA GCT CAA ATG CAA TG	59.0	
*Npy*	forward	CCA GGC AGA GAT ACG GCA AGA GAT C	60.7	119 bp
	reverse	CCA TCA CCA CAT GGA AGG GTC C	60.0	
*Per1*	forward	CTC TTC TTC TGG CAA TGG CAA GGA C	60.0	120 bp
	reverse	GCA CTC AGG AGG CTA TAG GCA ATG	59.4	
*Actb*	forward	ACC TCA TGA AGA TCC TGA CCG AGC	60.3	120 bp
	reverse	CCA TCT CTT GCT CGA AGT CCA GGG	61.1	
*Hprt*	forward	AGT CCC AGC GTC GTG ATT AGT GAT G	60.4	140 bp
	reverse	CGA GCA AGT CTT TCA GTC CTG TCC A	60.5	
*Rplp0*	forward	GCA ACA GTC GGG TAA CCA ATC TGC	60.4	153 bp
	reverse	CTTCGGGCTCATCATCCAGCAG	60.1	
*Rn18s*	forward	GCT CCT CTC CTA CTT GGA TAA CTG TG	59.2	126 bp
	reverse	CGG GTT GGT TTT GAT CTG ATA AAT GCA	59.4	

### Real-time qPCR (RT-qPCR) and analysis of expression data

Real-time qPCR was performed to compare relative gene expression values of investigated genes between and within SDT, FIT-SD, FIT-LD and the non-torpid control group over the course of a day and during different torpor stages.

RT-qPCR experiments were carried out on an ABI Prism 7300 Real Time PCR System (Applied Biosystems, Darmstadt, Germany) using Power SYBR® Green PCR Master Mix (Applied Biosystems, Darmstadt, Germany). Due to the large number of samples, RT-qPCRs were performed on five 96-well plates (Biolabproducts, Bebensee, Germany) for each target gene with a non-torpid ZT16 sample applied to all plates as inter-plate calibrator. *Hprt*, *Actb*, *Rplp0* and *Rn18s* were employed as putative non-regulated controls. Since none of these reference genes showed stability of expression values across all investigated states, we used *Hprt* as reference gene for NT-SD and SDT and *Rplp0* as reference gene for FIT-SD and FIT-LD. To calculate PCR efficiency, a series of six 10-fold dilutions of target gene specific standard plasmids was added to the plate from which standard curves were generated. Specificity of each amplification reaction was validated by dissociation curve analysis. All samples were run in triplicates, using 1 μl cDNA as template for each reaction. Furthermore, a no-template control was run on each plate in duplicates. RT-qPCR was conducted with a standard cycling protocol using 40 amplification cycles (50°C 2 min; 95°C 10 min; 95°C 15 s; 60°C 15 s; 72°C 30 s).

First evaluation of RT-qPCR data was done with the 7500 Software v2.0.6 (ABI Prism, Applied Biosystems). Afterwards RT-qPCR data were exported to Microsoft Excel 2010 to estimate expression levels of investigated genes using the ΔΔCT method. Differences in relative mRNA expression were assessed during torpor entrance, mid torpor, arousal and post torpor and are shown relative to NT-SD at same ZT respectively. Relative mRNA expression levels over the circadian cycle of investigated genes are shown for SDT, FIT-SD and NT-SD relative to the corresponding ZT1 sample of each group. Since FIT-LD was not sampled at specific ZT time points, but sampling was only defined by torpor state, this group was excluded from circadian rhythmicity analysis.

### Statistical analysis

All statistical analysis and figures were performed with SigmaPlot 12.5 (Systat Software Inc). Differences in torpor duration and depth between SDT, FIT-SD and FIT-LD were statistically tested by Mann Whitney Rank Sum test (U-test). The qPCR data were statistically tested by two-way ANOVA with the factors torpor group (SDT, FIT-SD, FIT-LD, NT-SD) and torpor state/time of day (torpor entrance, mid torpor, arousal, post torpor) followed by Tukey test or by one-way ANOVA or Kruskal-Wallis test, if normality test failed in at least one of the investigated groups. P-values ≤ 0.05 were considered as significant.

## Results

### Torpor depth and duration in fasting-induced and spontaneous daily torpor

**Fig 2:**
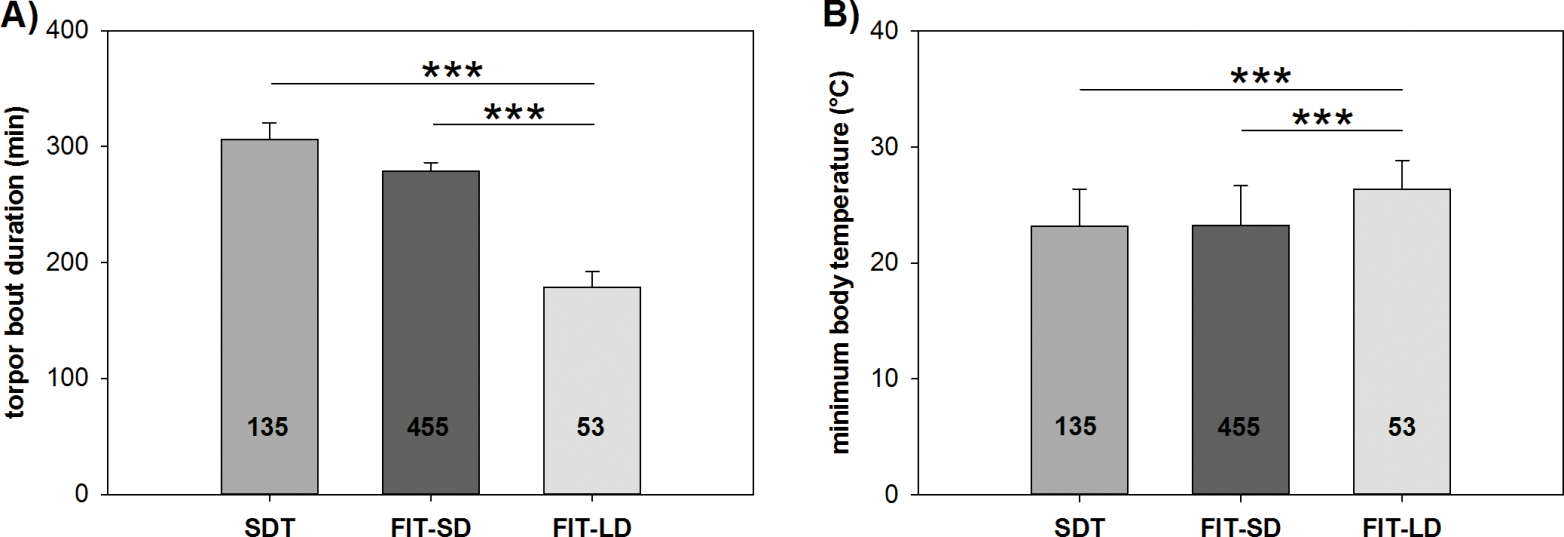
**Average torpor bout duration (A) and depth (B) of hamsters undergoing SDT, FIT-SD and FIT-LD.** Numbers within bars indicate the number of torpor episodes within each group used for statistical analysis. Significant differences between these three types of torpor are marked with * = p<0.05, ** = p<0.01 and *** = p<0.001.

We calculated the mean torpor duration and minimal T_b_ of hamsters undergoing SDT, FIT-SD and FIT-LD. Torpor was defined as T_b_ < 32°C for more than 30 minutes. Animals undergoing SDT had a mean torpor duration of 306.0 ± 14.46 minutes with a minimal T_b_ of 23.2 ± 0.27°C. FIT-SD animals showed a slightly lower mean torpor duration of 279.02 ±7.19 minutes and minimal T_b_ of 23.2 ± 0.16°C. FIT-LD animals showed significantly shorter torpor bouts with a mean torpor duration of 178.6 ± 13.53 minutes (SDT vs. FIT-LD: U-test, P<0.001; FIT-SD vs. FIT-LD: U-test, P<0.001) as well as a significantly higher minimal T_b_ of 26.3 ± 0.33°C (SDT vs. FIT-LD: U-test, P<0.001; FIT-SD vs. FIT-LD: U-test, P<0.001) compared to SDT and FIT-SD (Fig 2).

### Hypothalamic gene expression of genes involved in orexigenic, circadian and thyroid hormone regulatory mechanisms

To determine whether spontaneous daily torpor and fasting-induced torpor might underlie different regulatory systems, we investigated hypothalamic mRNA expression levels of *Npy*, *Agrp*, *Per1*, *Bmal1*, *Dio2* and *Mct8* as representatives for orexigenic, circadian and thyroid hormone regulatory mechanisms.

#### Relative mRNA expression of *Agrp* and *Npy* over the course of a torpor bout

**Fig 3:**
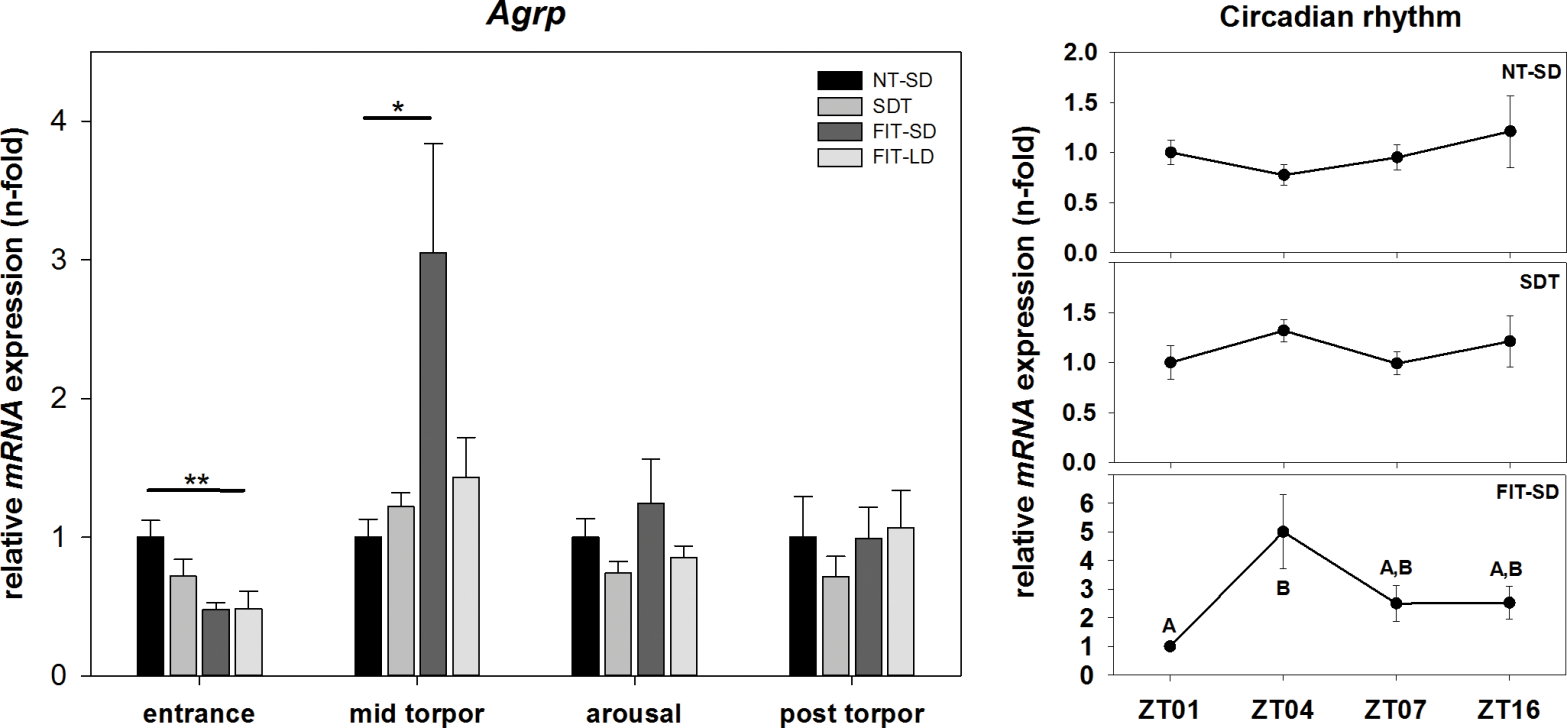
Relative mRNA expression of *Agrp*. Bar graphs (with n = 5 for each bar) show fold changes of mRNA expression for hamsters undergoing SDT (mid grey bars, ±SEM), FIT-SD (dark grey bars, ±SEM) and FIT-LD (light grey bars, ±SEM) relative to NT-SD (black bars, ±SEM) for torpor entrance (ZT1), mid torpor (ZT4), arousal (ZT7) and post torpor (ZT16) respectively. Significant differences within each torpor state are marked with * = p<0.05, ** = p<0.01 and *** = p<0.001. Line graphs show differences in mRNA expression (±SEM) at four different time points (with n = 5 for each time point) over the course of a day relative to ZT1 for hamsters remaining active (NT-SD), undergoing SDT or FIT-SD. Significant differences are marked with different upper cases (p<0.05).

There was an effect on *Agrp* mRNA expression in torpor group during torpor entrance (one-way ANOVA, p = 0.003) and mid torpor (one-way ANOVA, p = 0.034). During torpor entrance, *Agrp* mRNA expression was 0.52-fold down regulated in FIT-LD relative to NT-SD at ZT1 (FIT-LD vs. NT-SD: Tukey test, p = 0.002). During mid torpor, expression of *Agrp* in FIT-SD showed a 3.05-fold up regulation compared to NT-SD (FIT-SD vs. NT-SD: Tukey test, p = 0.034). SDT and FIT-LD showed no significant differences compared to NT-SD during mid torpor. There were no significant changes in *Agrp* expression during arousal or post torpor among all investigated torpor groups (Fig 3).

There was no effect of time of day or torpor state in *Agrp* expression for NT-SD (Kruskal-Wallis test, p = 0.574) and SDT (one-way ANOVA, p = 0.178). In FIT-SD there was an effect (one-way ANOVA, p = 0.034) showing elevated mRNA expression during mid torpor at ZT4 compared to torpor entrance at ZT1 (ZT4 vs. ZT1: Tukey test, p = 0.022) (Fig 3).

**Fig 4:**
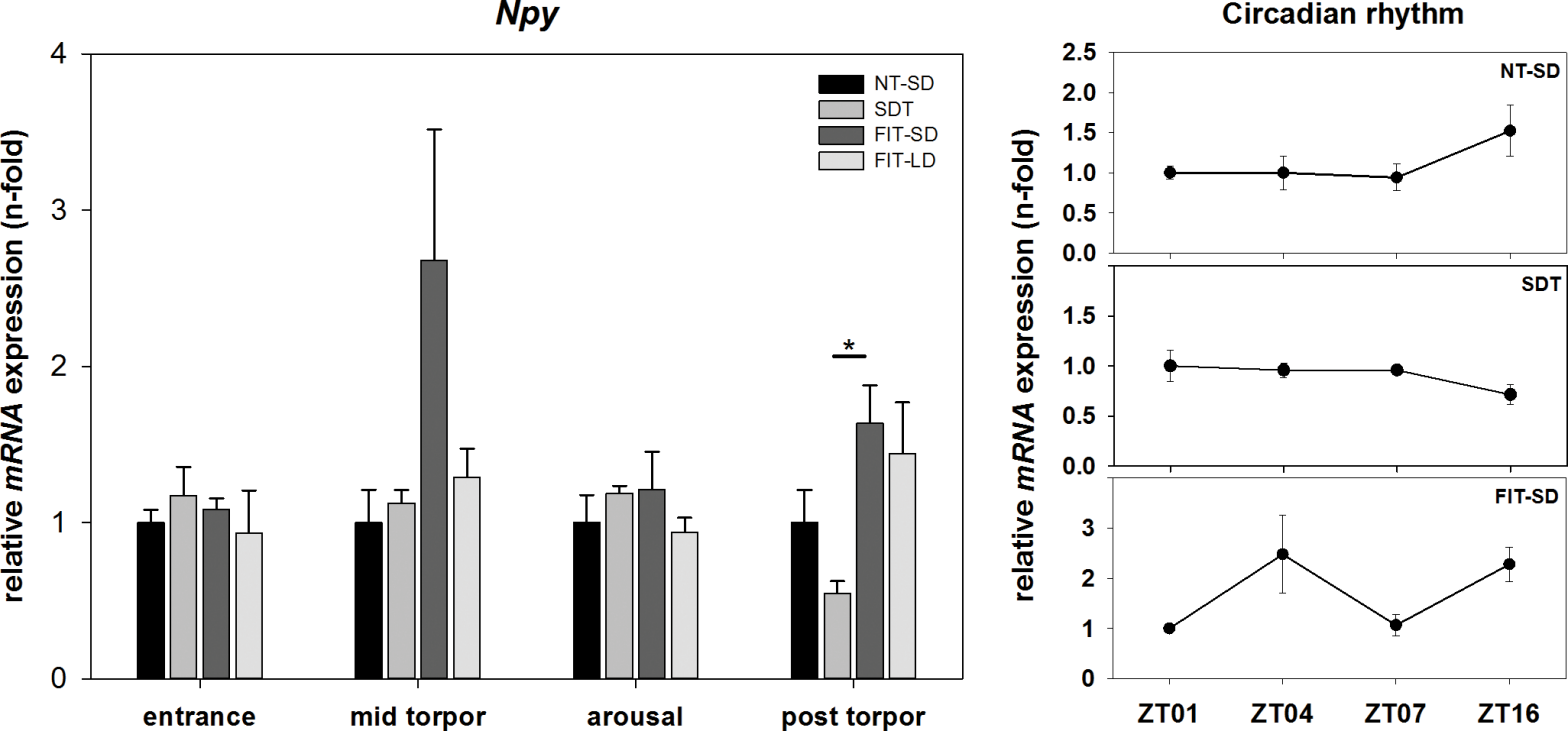
Relative mRNA expression of *Npy*. Bar graphs (with n = 5 for each bar) show fold changes of mRNA expression for hamsters undergoing SDT (mid grey bars, ±SEM), FIT-SD (dark grey bars, ±SEM) and FIT-LD (light grey bars, ±SEM) relative to NT-SD (black bars, ±SEM) for torpor entrance (ZT1), mid torpor (ZT4), arousal (ZT7) and post torpor (ZT16) respectively. Significant differences within each torpor state are marked with * = p<0.05, ** = p<0.01 and *** = p<0.001. Line graphs show differences in mRNA expression (±SEM) at four different time points (with n = 5 for each time point) over the course of a day relative to ZT1 for hamsters remaining active (NT-SD), undergoing SDT or FIT-SD. Significant differences are marked with different upper cases (p<0.05).

There was an effect of torpor group on *Npy* mRNA expression during the post torpid state (Kruskal-Wallis test, p = 0.045). qPCR analysis revealed no significant differences of *Npy* expression during torpor entrance, mid torpor or arousal between SDT, FIT-SD or FIT-LD. However, there was a trend towards up regulation in FIT-SD during mid torpor compared to NT-SD (FIT-SD vs. NT-SD: Tukey test, p = 0.090). Only during the post torpid state a significant difference could be found between SDT and FIT-SD (SDT vs. FIT-SD: Tukey test, p<0.05) (Fig 4).

There was no effect of time of day or torpor state in *Npy* expression for NT-SD (one-way ANOVA, p = 0.195), SDT (one-way ANOVA, p = 0.275) or FIT-SD (one-way ANOVA, p = 0.144) (Fig 4).

#### Relative mRNA expression of *Bmal1* and *Per1* over the course of a torpor bout

**Fig 5:**
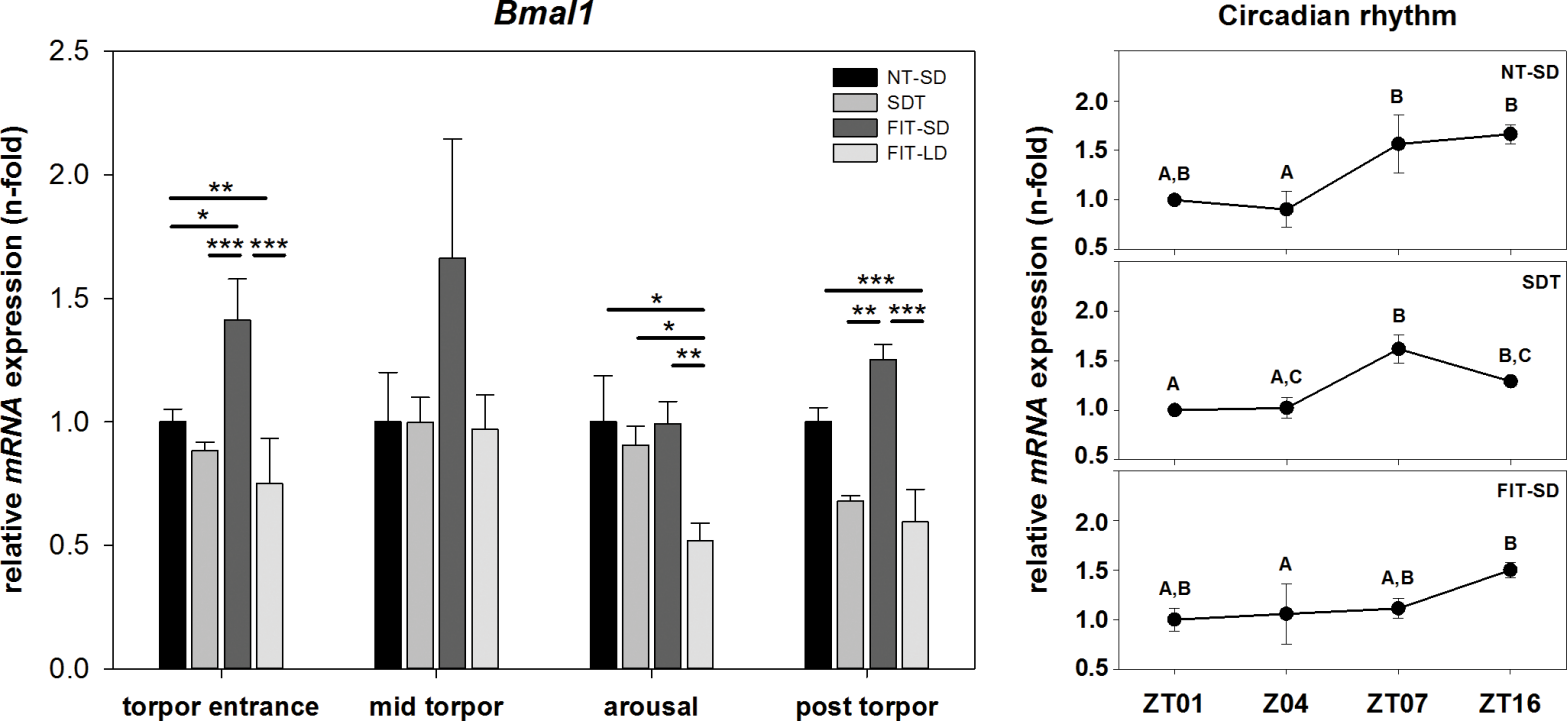
Relative mRNA expression of *Bmal1*. Bar graphs (with n = 5 for each bar) show fold changes of mRNA expression for hamsters undergoing SDT (mid grey bars, ±SEM), FIT-SD (dark grey bars, ±SEM) and FIT-LD (light grey bars, ±SEM) relative to NT-SD (black bars, ±SEM) for torpor entrance (ZT1), mid torpor (ZT4), arousal (ZT7) and post torpor (ZT16) respectively. Significant differences within each torpor state are marked with * = p<0.05, ** = p<0.01 and *** = p<0.001. Line graphs show differences in mRNA expression (±SEM) at four different time points (with n = 5 for each time point) over the course of a day relative to ZT1 for hamsters remaining active (NT-SD), undergoing SDT or FIT-SD. Significant differences are marked with different upper cases (p<0.05).

There was an effect of torpor group on *Bmal1* mRNA expression during torpor entrance (one-way ANOVA, p<0.001), arousal (one-way ANOVA, p = 0.002) and post torpor (one-way ANOVA, p<0.001), as well as of torpor state (NT-SD: one-way ANOVA, p = 0.002; SDT: one-way ANOVA, p = 0.003; FIT-SD: Kruskal-Wallis test, p = 0.024). During torpor entrance, *Bmal1* mRNA expression was 0.25-fold down regulated in FIT-LD compared to NT-SD (FIT-LD vs NT-SD: Tukey test, p = 0.003), but 1.41-fold up regulated in FIT-SD compared to NT-SD (FIT-SD vs NT-SD: Tukey test, p = 0.031) with a higher expression level compared to SDT and FIT-LD (FIT-SD vs. SDT: Tukey test, p<0.001; FIT-SD vs. FIT-LD: Tukey test, p<0.001). During arousal *Bmal1* in FIT-LD was 0.48-fold down regulated relative to NT-SD (FIT-LD vs. NT-SD: Tukey test, p = 0.012) and the expression level was significantly lower than in SDT (FIT-LD vs. SDT: Tukey test, p = 0.045) and FIT-SD (FIT-LD vs. FIT-SD: Tukey test, p = 0.001). In the post torpid state *Bmal1* expression was 0.41-fold down regulated in FIT-LD (FIT-LD vs. NT-SD: Tukey test, p<0.001). FIT-SD showed a significantly higher expression level than SDT (FIT-SD vs SDT: Tukey test, p = 0.003) and FIT-LD (FIT-SD vs. FIT-LD: Tukey test, p<0.001) (Fig 5).

There was an effect of time of day or torpor state in *Bmal1* expression. In NT-SD *Bmal1* expression was low at ZT1 and ZT4, significantly increased 1.56-fold at ZT7 and remained 1.66-fold up regulated at ZT16 (ZT1 vs. ZT16: Tukey test, p = 0.052; ZT4 vs. ZT7: Tukey test, p = 0.011; ZT4 vs. ZT16: Tukey test, p = 0.002). In SDT *Bmal1* expression was low at ZT1 and ZT4, increased 1.62-fold at ZT7 before slightly decreasing again to 1.29-fold at ZT16 (ZT1 vs. ZT7: Tukey test, p = 0.004; ZT1 vs. ZT16: Tukey test, p = 0.020; ZT4 vs. ZT7: Tukey test, p = 0.043). In FIT-SD *Bmal1* expression was low at ZT1, ZT4 (1.06-fold) and ZT7 (1.11-fold), but 1.50-fold up regulated at ZT16 (ZT4 vs. ZT16: Tukey test, p<0.05) (Fig 5).

**Fig 6:**
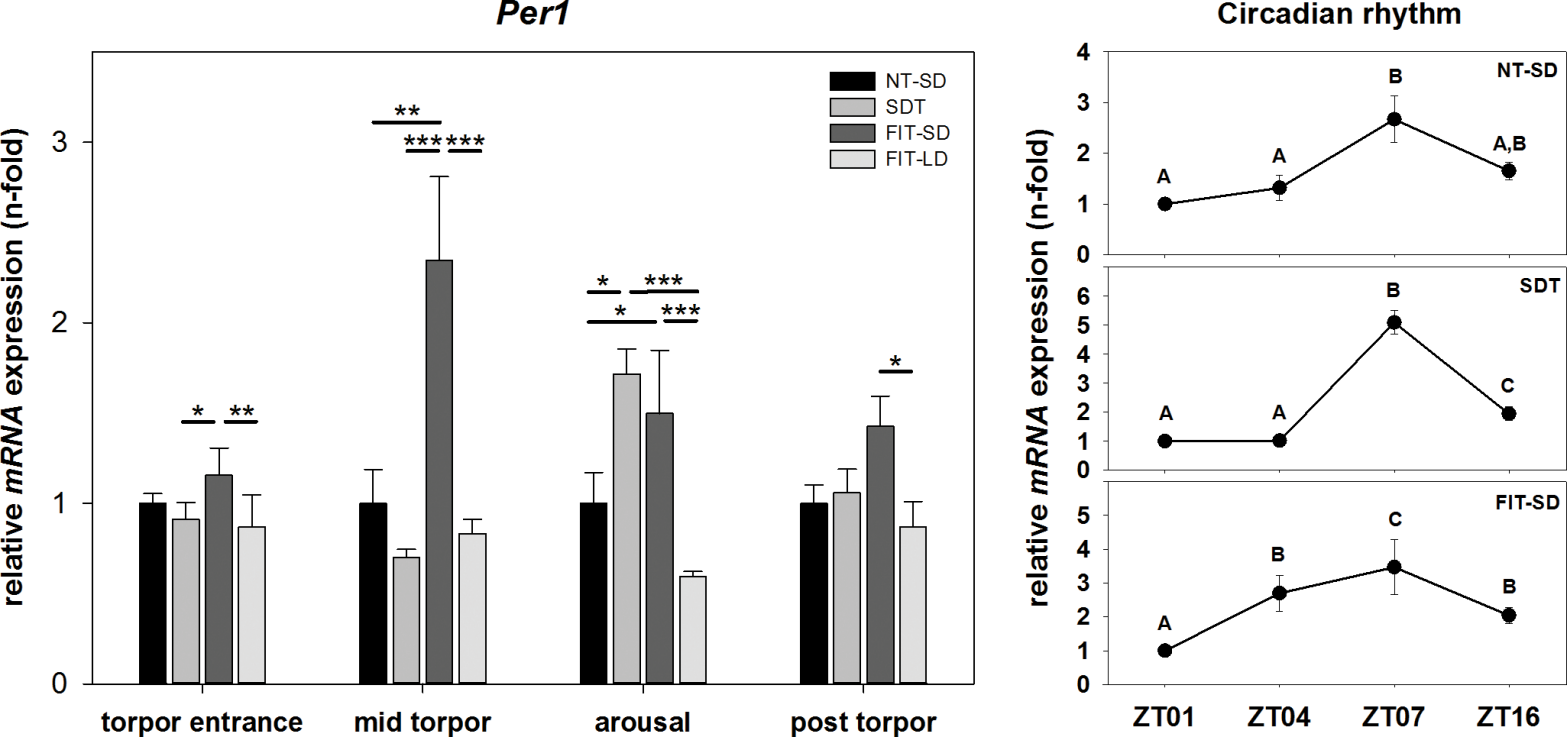
Relative mRNA expression of *Per1*. Bar graphs (with n = 5 for each bar) show fold changes of mRNA expression for hamsters undergoing SDT (mid grey bars, ±SEM), FIT-SD (dark grey bars, ±SEM) and FIT-LD (light grey bars, ±SEM) relative to NT-SD (black bars, ±SEM) for torpor entrance (ZT1), mid torpor (ZT4), arousal (ZT7) and post torpor (ZT16) respectively. Significant differences within each torpor state are marked with * = p<0.05, ** = p<0.01 and *** = p<0.001. Line graphs show differences in mRNA expression (±SEM) at four different time points (with n = 5 for each time point) over the course of a day relative to ZT1 for hamsters remaining active (NT-SD), undergoing SDT or FIT-SD. Significant differences are marked with different upper cases (p<0.05).

There was an effect of torpor state on *Per1* mRNA expression (two-way ANOVA, p<0.001) as well as of torpor group (two-way Anova, p<0.001) showing a significant interaction between torpor state and torpor group (two-way ANOVA, p = 0.002). During torpor entrance, *Per1* expression in FIT-SD showed a significantly higher value compared to SDT (FIT-SD vs. SDT: Tukey test, p = 0.010) and FIT-LD (FIT-SD vs. FIT-LD: Tukey test, p = 0.002). During mid torpor, *Per1* in FIT-SD was 2.35-fold up regulated relative to NT-SD (FIT-SD vs. NT-SD: Tukey test, p = 0.001) and was significantly higher expressed than in SDT (FIT-SD vs. SDT: Tukey test, p<0.001) and FIT-LD (FIT-SD vs. FIT-LD: Tukey test, p<0.001). During arousal, *Per1* expression was 1.72-fold up regulated in SDT (SDT vs. NT-SD: Tukey test, p = 0.012) and 1.50-fold up regulated in FIT-SD (FIT-SD vs. NT-SD: Tukey-test, p = 0.039) compared to NT-SD, with a significantly lower expression value of FIT-LD compared to SDT (FIT-LD vs. SDT: Tukey test, p<0.001) and FIT-SD (FIT-LD- vs. FIT-SD: Tukey test, p<0.001). In the post torpid state, *Per1* was significantly lower expressed in FIT-LD than in FIT-SD (FIT-LD vs. FIT-SD: Tukey test, p = 0.013) (Fig 6).

There was an effect of time of day or torpor state in *Per1* expression. In NT-SD *Per1* expression was slightly increased 1.32-fold at ZT4 and increased 2.67-fold at ZT7, before decreasing again to a fold change of 1.65 at ZT16 (ZT1 vs. ZT7: Tukey test, p<0.001; ZT4 vs. ZT7: Tukey test, p<0.001). In SDT *Per1* expression remained on a low expression level of 1.02-fold at ZT4, increased 5.09-fold at ZT7 and decreased again to a fold change of 1.94 at ZT16 (ZT1 vs. ZT7: Tukey test, p<0.001; ZT1 vs. ZT16: Tukey test, p<0.001; ZT4 vs. ZT7: Tukey test, p<0.001; ZT4 vs. ZT16: Tukey test, p = 0.002; ZT7 vs. ZT16: Tukey test, p<0.001). In FIT-SD *Per1* expression was 2.70-fold up regulated at ZT4, increased by 3.47-fold at ZT7 before decreasing to 2.04-fold at ZT16 (ZT1 vs. ZT4: Tukey test, p = 0.008; ZT1 vs. ZT7: Tukey test, p<0.001; ZT1 vs. ZT16: Tukey test, p = 0.020; ZT7 vs. ZT4: Tukey test, p = 0.013; ZT7 vs. ZT16: Tukey test, p = 0.005) (Fig 6).

#### Relative mRNA expression of *Dio2* and *Mct8* over the course of a torpor bout

**Fig 7:**
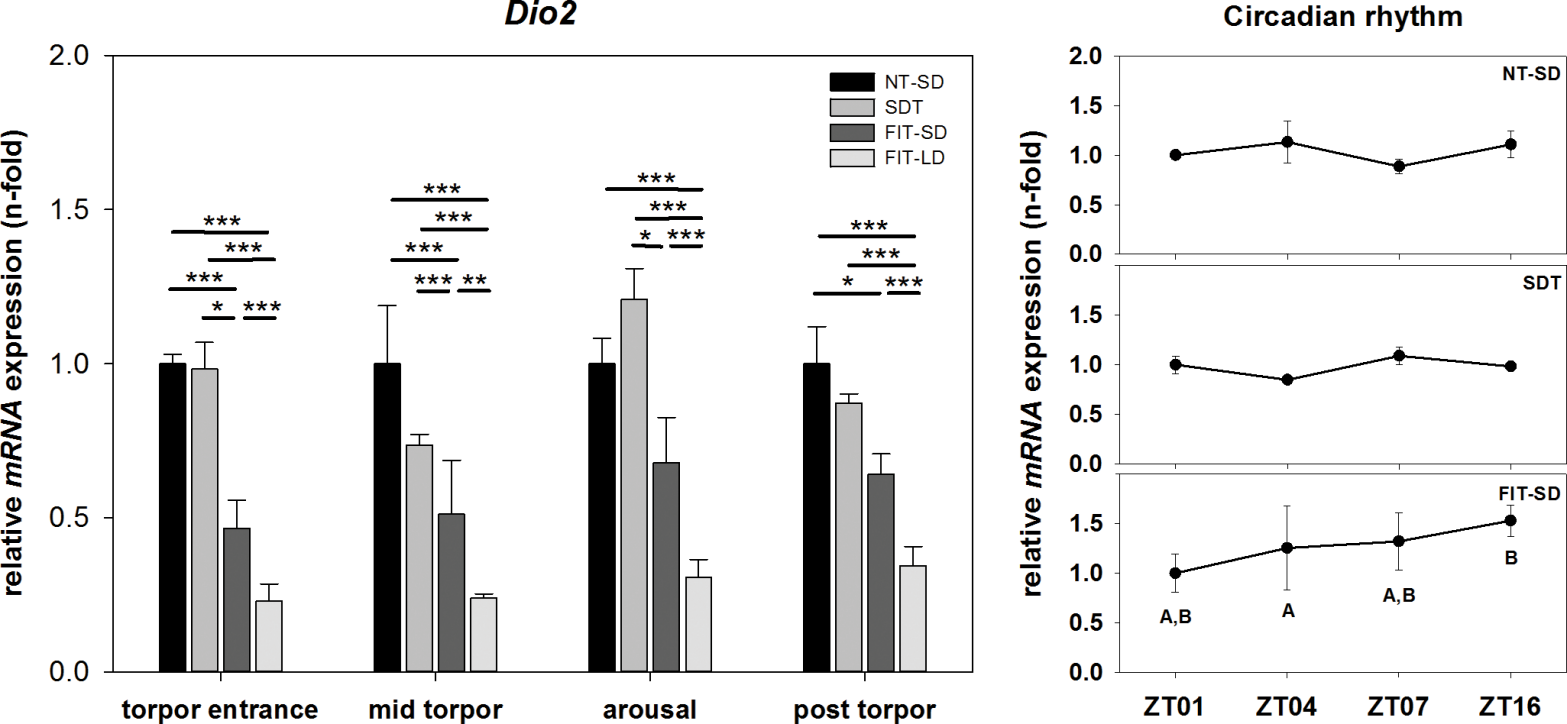
Relative mRNA expression of *Dio2*. Bar graphs (with n = 5 for each bar) show fold changes of mRNA expression for hamsters undergoing SDT (mid grey bars, ±SEM), FIT-SD (dark grey bars, ±SEM) and FIT-LD (light grey bars, ±SEM) relative to NT-SD (black bars, ±SEM) for torpor entrance (ZT1), mid torpor (ZT4), arousal (ZT7) and post torpor (ZT16) respectively. Significant differences within each torpor state are marked with * = p<0.05, ** = p<0.01 and *** = p<0.001. Line graphs show differences in mRNA expression (±SEM) at four different time points (with n = 5 for each time point) over the course of a day relative to ZT1 for hamsters remaining active (NT-SD), undergoing SDT or FIT-SD. Significant differences are marked with different upper cases (p<0.05).

There was an effect of torpor state on *Dio2* mRNA expression (two-way ANOVA, p = 0.002) as well as of torpor group (two-way ANOVA, p<0.001) showing no significant interaction between torpor state and torpor group. During torpor entrance, *Dio2* mRNA expression in FIT-SD was 0.53-fold down regulated compared to NT-SD (FIT-SD vs. NT-SD: Tukey test, p<0.001) and was significantly lower than in SDT (FIT-SD vs. SDT: Tukey test. p = 0.015). FIT-LD showed a 0.77-fold down regulation compared to NT-SD (FIT-LD vs. NT-SD: Tukey test, p<0.001) and had a significantly lower expression level compared to SDT (FIT-LD vs. SDT: Tukey test, p<0.001) and FIT-SD (FIT-LD vs. FIT-SD: Tukey test, p<0.001). During mid torpor, FIT-SD was 0.49-fold down regulated compared to NT-SD (FIT-SD vs. NT-SD: Tukey test, p<0.001) and *Dio2* expression was significantly lower than in SDT (FIT-SD vs. SDT: Tukey test, p<0.001). FIT-LD was 0.76-fold down regulated compared to NT-SD (FIT-LD vs NT-SD: Tukey test, p<0.001) with reduced expression in FIT-LD compared to SDT (FIT-LD vs. SDT: Tukey test, p<0.001) and FIT-SD (FIT-LD vs. FIT-SD: Tukey test, p = 0.002). During arousal, *Dio2* expression was significantly lower in FIT LD than in NT-SD (FIT-LD vs. NT-SD: Tukey test, p<0.001), SDT (FIT-LD vs. SDT: Tukey test, p<0.001) and FIT-SD (FIT-LD vs. FIT-SD: Tukey test, p<0.001) with a fold change of 0.31 relative to NT-SD. *Dio2* expression was significantly lower in FIT-SD than in SDT (FIT-SD vs. SDT: Tukey test, p = 0.027). In the post torpid state, *Dio2* expression of FIT-SD was 0.36-fold down regulated relative to NT-SD (FIT-SD vs. NT-SD: Tukey test, p = 0.038). Lowest expression level was shown by FIT-LD with a fold change of 0.35 (FIT-LD vs NT-SD: Tukey test, p<0.001; FIT-LD vs. SDT: Tukey test, p<0.001; FIT-LD vs. FIT-SD: Tukey test, p<0.001) (Fig 7).

There was no effect of time of day or torpor state in *Dio2* expression for NT-SD and SDT but there was an effect in *Dio2* expression for FIT-SD. During FIT-SD mRNA expression was elevated post torpor at ZT16 compared to mid torpor at ZT4 (ZT16 vs. ZT4: Tukey test, p = 0.012) (Fig 7).

**Fig 8:**
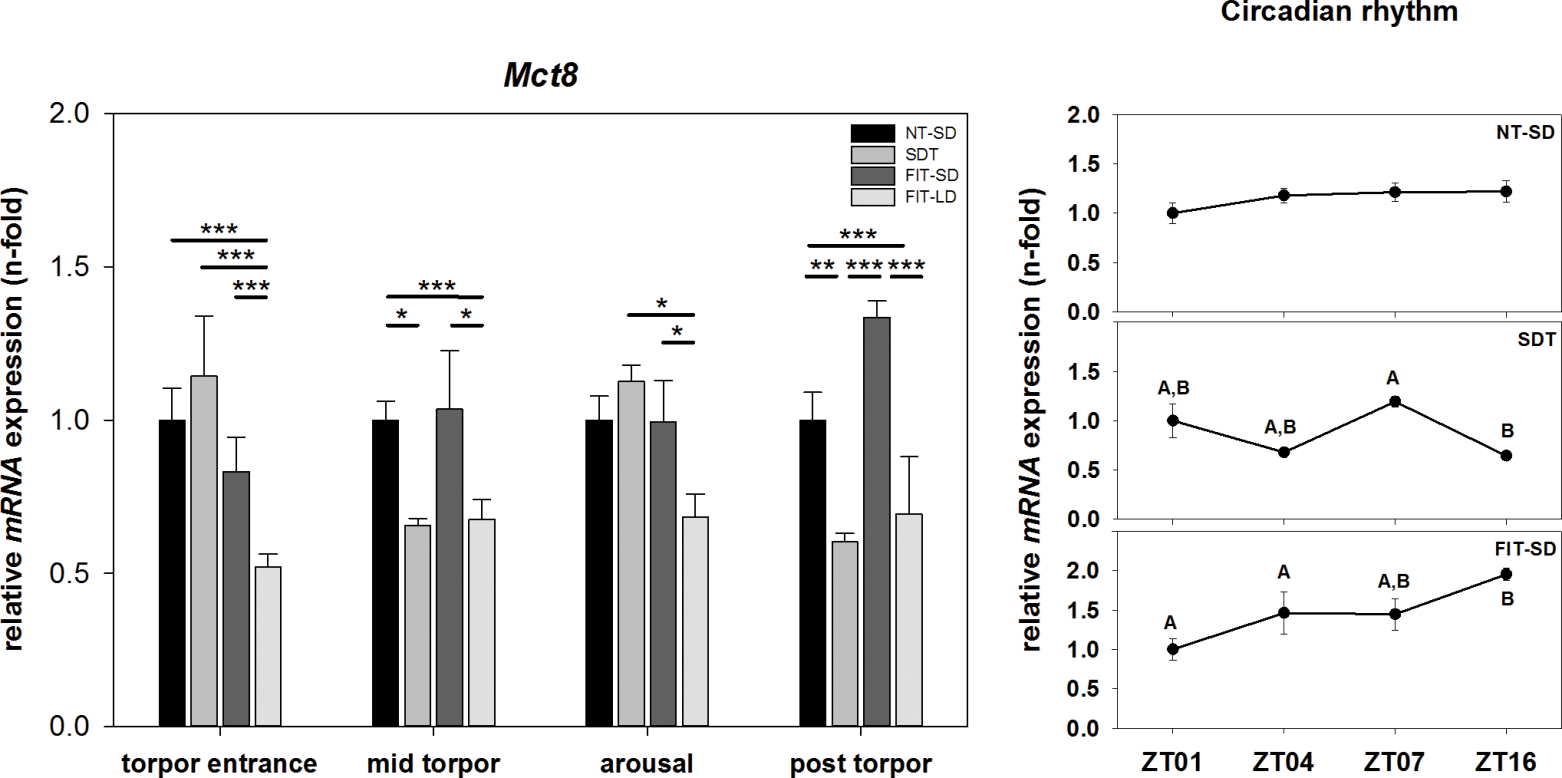
Relative mRNA expression of *Mct8*. Bar graphs (with n = 5 for each bar) show fold changes of mRNA expression for hamsters undergoing SDT (mid grey bars, ±SEM), FIT-SD (dark grey bars, ±SEM) and FIT-LD (light grey bars, ±SEM) relative to NT-SD (black bars, ±SEM) for torpor entrance (ZT1), mid torpor (ZT4), arousal (ZT7) and post torpor (ZT16) respectively. Significant differences within each torpor state are marked with * = p<0.05, ** = p<0.01 and *** = p<0.001. Line graphs show differences in mRNA expression (±SEM) at four different time points (with n = 5 for each time point) over the course of a day relative to ZT1 for hamsters remaining active (NT-SD), undergoing SDT or FIT-SD. Significant differences are marked with different upper cases (p<0.05).

There was an effect of torpor state on *Mct8* mRNA expression (two-way ANOVA, p<0.001) as well as of torpor group (two-way ANOVA, p<0.001) showing a significant interaction between torpor state and torpor group (two-way ANOVA, p<0.001). During torpor entrance, *Mct8* mRNA expression of FIT-LD was 0.48-fold down regulated and differed significantly from NT-SD (FIT-LD vs NT-SD: Tukey test, p<0.001), SDT (FIT-LD vs. SDT: Tukey test, p<0.001) and FIT-SD (FIT-LD vs. FIT-SD: Tukey test, p<0.001). During mid torpor, *Mct8* expression was 0.34-fold down regulated in SDT (SDT vs. NT-SD: Tukey test, p = 0.044). FIT-LD was 0.32-fold down regulated (FIT-LD vs. NT-SD: Tukey test, p<0.001) and was significantly lower expressed than in FIT-SD (FIT-LD vs. FIT-SD: Tukey test, p = 0.011). During arousal, *Mct8* expression value was significantly lower in FIT-LD than in SDT (FIT-LD vs. SDT: Tukey test, p = 0.018) and FIT-SD (FIT-LD vs. FIT-SD: Tukey test, p = 0.012). In the post torpid state, *Mct8* was 0.40-fold down regulated in SDT and differed significantly from NT-SD (SDT vs. NT-SD: Tukey test, p = 0.006) and FIT-SD (SDT vs. FIT-SD: Tukey test, p<0.001). *Mct8* expression in FIT-LD was 0.31-fold down regulated and differed significantly from NT-SD (FIT-LD vs. NT-SD: Tukey test, p<0.001) and FIT-SD (FIT-LD vs. FIT-SD: Tukey test, p<0.001) (Fig 8).

There was no effect of time of day or torpor state in *Mct8* mRNA expression for NT-SD, but there was an effect of torpor state in SDT and FIT-SD. In SDT *Mct8* mRNA expression during arousal at ZT7 was higher than in the post torpid state at ZT16 (ZT7 vs. ZT16, p = 0.002). *Mct8* expression in FIT-SD was elevated in the post torpid state at ZT16 compared to torpor entrance at ZT1 and mid torpor at ZT4 (ZT1 vs. ZT16: Tukey test, p = 0.001; ZT4 vs. ZT16: Tukey test, p<0.001) (Fig 8).

## Discussion

In this study, we investigated differences between spontaneous daily torpor and fasting-induced torpor on the physiological and molecular levels in the Djungarian hamster. As previously shown, FIT-LD bouts differed from SDT in depth and duration. In FIT-LD bouts, the plateau phase of torpor maintenance, with almost constant low metabolic rate, is missing. This leads to shorter and shallower torpor episodes, consisting of torpor entrance directly followed by arousal. The mid torpor phase however, is mainly responsible for the energy savings accrued during torpor. Therefore single FIT bouts are less effective in saving energy than SDT bouts. The lower energy savings achieved from FIT bouts can be compensated by multiple FIT bouts per day [19,20,44]. In our study, no significant difference in depth or duration between FIT-SD and SDT in winter adapted animals was apparent. This is in agreement with earlier findings, showing differences in torpor depth and duration only between SDT and FIT-LD but not for SDT and FIT-SD. However, there was a higher mean torpor incidence of animals expressing FIT-SD compared to SDT [19]. Hence, winter adapted hamsters seem to increase the torpor frequency to increase energy saving, whereas summer adapted hamsters are able to use multiple torpor episodes per day to adjust energy requirements.

To dissect out differences in potential control mechanisms of FIT and SDT, we investigated differential gene expression in hypothalamic regulatory centers involved in the control of acute and seasonal energy balance over the course of a torpor bout.

Expression analysis of *Agrp* and *Npy* showed dissimilar mRNA expression patterns between SDT and FIT-SD. *Agrp* and *Npy* are up regulated when leptin and insulin actions are decreased in the hypothalamus to stimulate food intake and reduce energy expenditure [21,23,45].

During SDT these two neuropeptides displayed no elevated mRNA expression levels in either of the investigated torpor states. This suggests that hamsters do not experience hunger before, during or after a spontaneously occurring torpor bout and indicates a balanced energy homeostasis that is still maintained while expressing SDT. Thus, no obvious energetic deficit appears to influence whether an animal enters torpor on that particular day or remains active. This is in accordance with physiological data, showing that spontaneous torpor is entered from a state of glucose metabolism (RQ ~1), hence metabolic balance [19].

Animals undergoing FIT-SD showed significantly up regulated *Agrp* expression during mid torpor and also *Npy* expression was up regulated during this state, although not reaching significance. *Npy* expression was also up regulated during the post torpid state of fasted hamsters. The up regulated mRNA levels of *Agrp* and *Npy* during FIT-SD point towards an acute negative energy balance in FIT expressing animals. This is in accordance with the observation that hamsters using FIT already exhibit a low respiratory quotient (0.79 ± 0.01) when entering the torpid state indicating a lipid-based metabolism, hence negative energy balance [19]. Also the increased *Npy* expression at night possibly indicates a state of hunger, when fasted animals are active again. The differences in *Agrp* and *Npy* expression between SDT and FIT were smaller than expected. Since whole hypothalamus was used for gene expression analysis, it might be possible that differences appear less pronounced than in specific nucleus analysis, caused by the signal to noise ratio. However, the negative energy balance caused by reduced food supply in SD animals seems to predominantly be compensated by an elevated torpor frequency rather than a change in single torpor bout characteristics *per se*. Since we only sampled the FIT-SD group after at least two days of food restriction, to ensure FIT expression, the hamsters were already able to adjust their torpor frequency to maintain energy balance, which is possibly reflected in relatively balanced *Agrp* and *Npy* expression.

Interestingly, we were not able to detect altered expression patterns of *Agrp* or *Npy* within the FIT-LD group. This could result from the extremely long fasting period, which was required to induce torpor in the obese LD adapted animals. A comparative study investigating the effect of acute food deprivation and chronic food restriction on peptides regulating food consumption, has shown that acute food deprivation leads to elevated *Agrp* expression in the hypothalamus of rats, whereas in chronically food restricted animals *Agrp* expression remained unaffected [46]. The same study found up regulation of *Npy* caused by both, acute food deprivation and chronic food restriction, however, with a significantly lower extent of up regulation in chronically food restricted rats [46]. In this approach, rats were fasted for 14 days to reach a chronically food restricted state, which is way shorter than the food restriction of over one month in our study. The extremely long period of food restriction could be the reason why we did not observe any alterations in *Npy* expression within the FIT-LD group. It is also possible that the long fasting period and accompanying body weight loss led to a decrease in energy requirements, so that 60% food supply was sufficient to maintain energy homeostasis.

No circadian rhythm was found in either, *Npy* or *Agrp* expression. This is accordance with *in-situ* hybridization data of Ellis et al. [47] who did not show a circadian rhythm for these genes in long or short photoperiod either. The difference observed for *Agrp* expression in FIT-SD between ZT1 and ZT4 reflects the torpid state rather than a circadian regulation.

*Bmal1* and *Per1* are parts of the molecular circadian clockwork. *Bmal1* acts as positive regulator in the circadian feedback loop and interacts with CLOCK to activate transcription of *Per* and *Cry* genes, whereas *Per1* is considered as negative regulator, inhibiting its own transcription by building a repressor complex of the PER1 protein together with CRY, which translocate to the nucleus and inhibits the CLOCK:BMAL1 complex [27].

Our results showed a circadian rhythm of *Bmal1* and *Per1* mRNA expression during SDT. As previously described, this indicates that the circadian clockwork is not disrupted during daily torpor and ensures the proper timing of SDT into the animal´s resting phase [13,29,30,48]. Some differences however, could be observed in clock gene expression between SDT and NT-SD, suggesting at least a modulatory effect of daily torpor. Compared to NT-SD, expression of *Per1* was significantly upregulated at ZT7, *Bmal1* expression already started to decrease at ZT16, whereas the expression level of *Bmal1* still remains high at ZT16 in the NT-SD group. The higher level of *Per1* combined with the earlier decrease of *Bmal1* during SDT could lead to a shortened free-running period in torpid animals that has previously been described for Djungarian hamsters undergoing torpor [49]. A direct effect of SDT on the circadian clock has previously been demonstrated, indicating alterations in phase and amplitude of the circadian clock during SDT [29].

Expression of these two clock genes during FIT-SD was clearly different from SDT and NT-SD. *Per1* expression peak was advanced whereas *Bmal1* was constantly low expressed throughout the torpid state with a slight up regulation at ZT16. Hence, the circadian feedback loop appears to be shifted in fasted hamsters. Fasting in general leads to an increased SIRT1 deacetylase activity [50,51]. SIRT1 is able to deacetylate BMAL1, which in turn can inactivate the BMAL1:CLOCK complex resulting in a disruption of the normal circadian feedback loop [52–54]. Since it is known that the major exogenous Zeitgeber for FIT expression is the feeding schedule rather than the light-dark cycle [30,55], it is likely that the circadian rhythm in FIT expressing animals was synchronized with the feeding schedule.

The FIT-LD group was not sampled at specific ZTs so that we were not able to investigate circadian rhythms in long term fasted hamsters.

*Dio2* showed decreased expression throughout the torpor bout in animals undergoing FIT-SD and FIT-LD. *Dio2* was also lower during mid torpor in SDT, but did not reach significance, most likely caused by the high standard error of the NT-SD group. The decreased *Dio2* expression observed in our study suggests a reduced conversion of T4 into the active metabolite T3 and would thereby cause low T3 availability in the hypothalamus during torpor. This is in accordance with earlier studies, demonstrating that high T3 levels specifically in the hypothalamus are able to block torpor in Djungarian hamsters, whereas systemically low T3 concentrations increase torpor frequency, depth and duration [40–42]. Here, we confirm that a lowered *Dio2* expression and potential decrease in local T3 availability appears to be a permissive factor for both, SDT as well as FIT in summer and winter adapted animals. Although starvation of hamsters has previously been shown to increase *Dio2* expression of SD adapted animals, we did not observe an up regulation in food restricted hamsters. In the previous study, however, hamsters were starved for 48 hours and killed in a non-torpid state [35], whereas animals in the current study still received 60% of their daily food consumption for more than two days. The differences in metabolic state and food supply may cause the diverging expression patterns.

Another factor which could influence the local thyroid hormone status of torpid Djungarian hamsters in hypothalamic neurons is the transporter protein *Mct8*. Our data showed decreased *Mct8* expression during mid torpor and in the post torpid state in SDT hamsters. Decreased expression of *Mct8* could support low T3 availability in the hypothalamus by reducing the thyroid hormone transport and possibly, together with the low *Dio2* mRNA level, facilitate the occurrence of torpor. This however, only seems to be true for SDT, since *Mct8* in FIT-SD animals showed no alteration relative to NT-SD during the torpor bout. Nevertheless, a number of different transporters, like organic anion transporters and L-type amino acid transporters, with the potential to transport thyroid hormones, have been identified and could affect the thyroid hormone status [56,57].

It has been shown that *Mct8* expression is higher in winter adapted animals compared to animals in summer state [35]. This fits with our observation of the overall decreased *Mct8* mRNA level within the FIT-LD group for all investigated torpor states, which is more likely caused by the animals’ seasonal state than by torpor state.

No circadian rhythm was found in either, *Dio2* or *Mct8* mRNA expression within the NT-SD groups. The isolated differences observed for *Dio2* expression in FIT-SD and for *Mct8* in SDT and FIT-SD seem to reflect differences caused by the torpid state rather than a circadian regulation.

Taken together, our data do not clearly support the hypothesis that SDT and FIT represent distinct physiological states. Although the circadian system is differentially regulated in the different torpor forms, gene expression changes in the orexigenic system only partly reflect the physiological data. The thyroid hormone system rather appears to be regulated by torpor *per se*, irrespective of the torpor form used. Studies on single hypothalamic nuclei including examination on protein level might better disentangle the regulatory mechanisms of the two torpor forms. However, the form of torpor used should be carefully taken in consideration when investigating and interpreting the phenomenon of torpor and its underlying mechanisms.
